# Oligometastatic esophageal cancer cured by systemic therapy combined with radiotherapy to primary tumor and metastasis (metastasis-directed therapy)—small case series

**DOI:** 10.37349/etat.2024.00255

**Published:** 2024-07-26

**Authors:** Mohan Hingorani, Hannah Stubley

**Affiliations:** University of Campania “Luigi Vanvitelli”, Italy; ^1^Department of Clinical Oncology, Hull University Teaching Hospitals NHS Trust, HU16 5JQ Hull, UK; ^2^Hull and York Medical School, YO10 5DD Hull, UK

**Keywords:** Oligometastasis, esophageal cancer, radiotherapy

## Abstract

The prognosis of metastatic esophageal cancer (EC) remains poor with an average life expectancy of around 9–12 months with standard systemic chemotherapy. The concept of oligometastatic disease (OMD) in EC cancer is controversial with no universally accepted definition. From the original cohort of metastatic oesophago-gastric (OG) cancer patients, 4 cases were identified that developed unusually favourable outcome with long-term survival and probable cure. In retrospect, all patients had OMD at presentation with striking similarities in terms of their clinical presentation, staging, treatment response and outcomes. All patients presented with locally advanced EC and 1–2 areas of metastatic disease (bone, lung, non-regional lymph node (LN) involvement). All were treated with combined therapeutic strategy using initial systemic chemotherapy followed by local radiotherapy to primary tumor and adjacent areas of visible/residual metastatic disease (metastasis-directed therapy). All patients experienced long-term survival (range = 7–13 years) with no evidence of recurrence and probable cure. The present case series adds to the growing pool of evidence indicating OM EC cancer represents a distinct and prognostically favorable subgroup.

## Introduction

The prognosis of metastatic esophageal cancer (EC) remains poor with an average life expectancy of around 9–12 months with standard systemic chemotherapy [1, 2]. The concept of oligometastatic disease (OMD) was first introduced in 1995 by Hellman and Weichselbaum and described as a stage of transition between localized and widespread metastatic disease [3]. It represents a probable stage in natural evolution of cancer in which tumor cells may proliferate in ‘sanctuaries’ hidden from systemic circulation before wider systemic dissemination. It remains unclear if there is a generic intermediate state of limited metastatic disease present in all cancer types or represents a more specific state driven by a particular molecular signature.

The concept of OMD in EC is controversial with no universally accepted definition. In a systematic review aimed at determining consensus definition OMD was commonly defined as involvement of 1 organ with less than 3 metastatic lesions or 1 extra-regional lymph node (LN) station [4].

We present small case series (*n* = 4) with advanced metastatic EC who developed an unusual and rather intriguing treatment response to standard therapy with long-term survival and probable cure. In retrospect, all the cases would have been classified as OM with striking similarities in terms of their clinical presentation and long-term outcomes. The primary intention of the manuscript is to highlight the probable presence of OMD state in EC and importance of the combined approach of local and metastasis-directed therapy.

## Case report

### Case selection (inclusion and exclusion criteria)

We previously reported on outcomes of patients with metastatic esophago-gastric cancer who were treated with chemotherapy followed by palliative radiotherapy (RT) to the primary tumor in the context of well-controlled metastatic disease. The median OS for patients treated with combined therapy (RT to primary tumor after initial chemotherapy) was 23.3 months [95% confidence interval (CI), 17.70 to 28.89 months] and significantly higher than the 14 months (95% CI, 10.91–17.08 months) in patients treated with chemotherapy alone (*P* < 0.001). RT was also associated with reduced incidence of stent insertion and tumor-related complications (e.g., bleeding, obstruction, etc.) [5].

From the original cohort of metastatic esophago-gastric cancer patients, we identified 4 cases who experienced exceptional outcomes with long-term survival and cure. All patients were diagnosed with metastatic oesophago-gastric (OG) cancer between years of February 2010 and June 2015. All patients had cancers of the esophagus and/or gastro-esophageal junction and had been treated with multi-modality therapy [initial chemotherapy followed by consolidation RT to site of primary tumor and adjacent areas of residual metastatic disease (metastasis-directed therapy)]. We selected patients for whom all relevant details of treatment delivered and long-term data of follow-up and survival were available. Patients with gastric cancer and those with incomplete follow-up data were excluded. These patients had striking similarities in terms of their clinical behavior, treatment responses and long-term outcomes. They experienced long-term survival consistent with probable cure. It became apparent that in hindsight these patients harbored OMD which may have contributed to the positive outcomes.

We interrogated the electronic records of above patients to identify different study parameters including clinical presentation, staging, treatment, follow up and long-term outcomes. Patients clinical stage has been defined using the American Joint Committee on Cancer (AJCC) version 9 classification system for the purpose of the manuscript. Subsequently, we screened the literature to identify the key evidence regarding management of OMD in esophago-gastric cancer.

### Patient (and treatment)—related characteristics and long-term outcomes

All patients were males with age ranging from 49–68 years and presented with dysphagia. They were fit and healthy for their age with no significant medical comorbidities and World Health Organisation (WHO) performance status (PS) score of 0 (independent, self-caring and normal levels of physical activity). All patients had an oral gastroduodenoscopy (OGD) as an initial investigation which identified the esophageal malignancy [lower/gastroesophageal junction (GOJ) = 3; middle = 1] and biopsies were obtained which revelated adenocarcinoma (AC) in 3 patients (cases 1–3) and squamous cell carcinoma (SqCC) in 1 patient (case 4). In addition, one patient (case 2) had evidence of Her-2 overexpression.

All patients underwent computed tomography (CT) and fluoro-deoxyglucose (FDG)-positron emission tomography (PET) staging scans and one patient also underwent endoscopic ultrasound (EUS) to determine the nature of para-esophageal lymphadenopathy. All patients were deemed to be metastatic/incurable after discussion in the multidisciplinary team (MDT) meeting attended by clinical oncologist, upper gastrointestinal surgeon, gastroenterologist, radiologist, and clinical nurse specialist.

The patterns of disease distribution and staging classification were as follows:

Case 1: multiple loco-regional LNs, para-aortic LN; bilateral lung nodules (T3N3M1);

Case 2: loco-regional gastro-hepatic node, para-aortic lymphadenopathy (T3N1M1);

Case 3: avid high left para-tracheal and T9 vertebral metastasis (T3N1M1);

Case 4: avid high paraesophageal LN (above arch aorta) (T3N0M1).

All patients had low volume 1–2 sites of metastatic disease including non-regional LN, bone and lung metastasis. Case 4 had an isolated high paraesophageal LN which was felt to be a non-regional LN outside of the surgical resection field (above arch of aorta) by the surgical team and the length of tumor was too long for definitive chemoradiotherapy.

All patients received first-line platinum-based chemotherapy most commonly with EOX (epirubicin, oxaliplatin, and capecitabine) regime [6 cycles (*n* = 2); 5 cycles (*n* = 1)] using EOX. Case 2 with Her-2 overexpression received 6 cycles of cisplatin, capecitabine and trastuzumab. All patients underwent CT after 3 and 6 cycles which showed responding/stable disease and all patients proceeded to consolidation RT using 30 Gy in 10 fractions to the main tumor with inclusion of any adjacent areas of metastatic disease that could be safely encompassed in a radiation field (metastasis-directed therapy). No further chemotherapy was given during or after RT apart from case 2 who continued with maintenance trastuzumab for total duration of 50 months when it was discontinued in view of cardiotoxicity.

### RT technique

**Figure 1:**
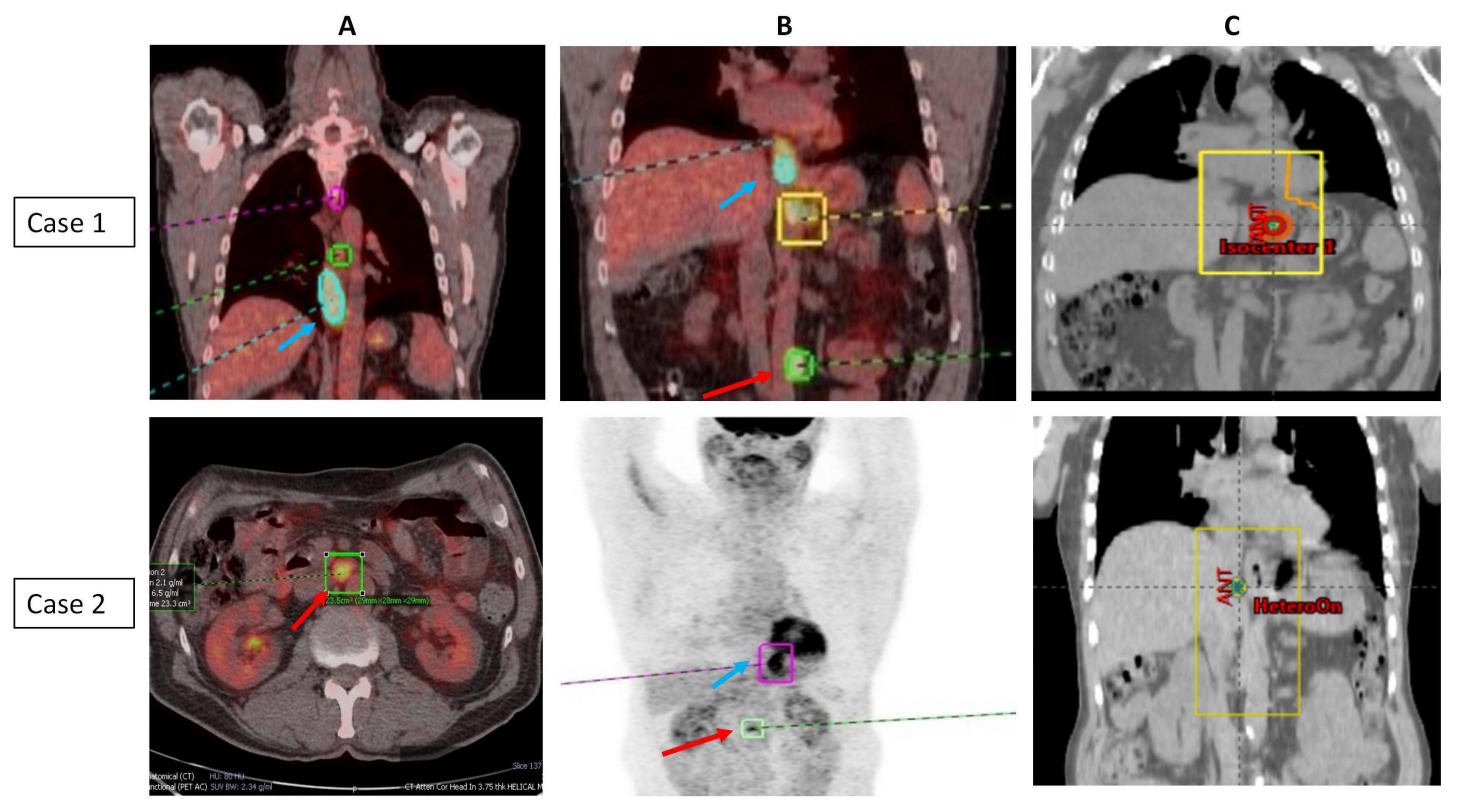
PET imaging showing the primary tumor and area of oligometastasis and corresponding radiotherapy portals (case 1 and case 2). (A) Coronal and axial PET scans of case 1 and case 2; (B) coronal PET scans of case 1 and case 2; (C) corresponding radiotherapy portals in case 1 and case 2. PET images with blue arrow indicating primary tumor and red arrow indicates site of oligometastasis [case 1: para-aortic node (patient also had lung metastasis)—both resolved completely after initial chemotherapy; case 2: paraaortic node]; radiotherapy portals were defined to cover the primary tumor and areas of adjacent metastatic disease/visible residual disease (metastasis-directed therapy)

**Figure 2:**
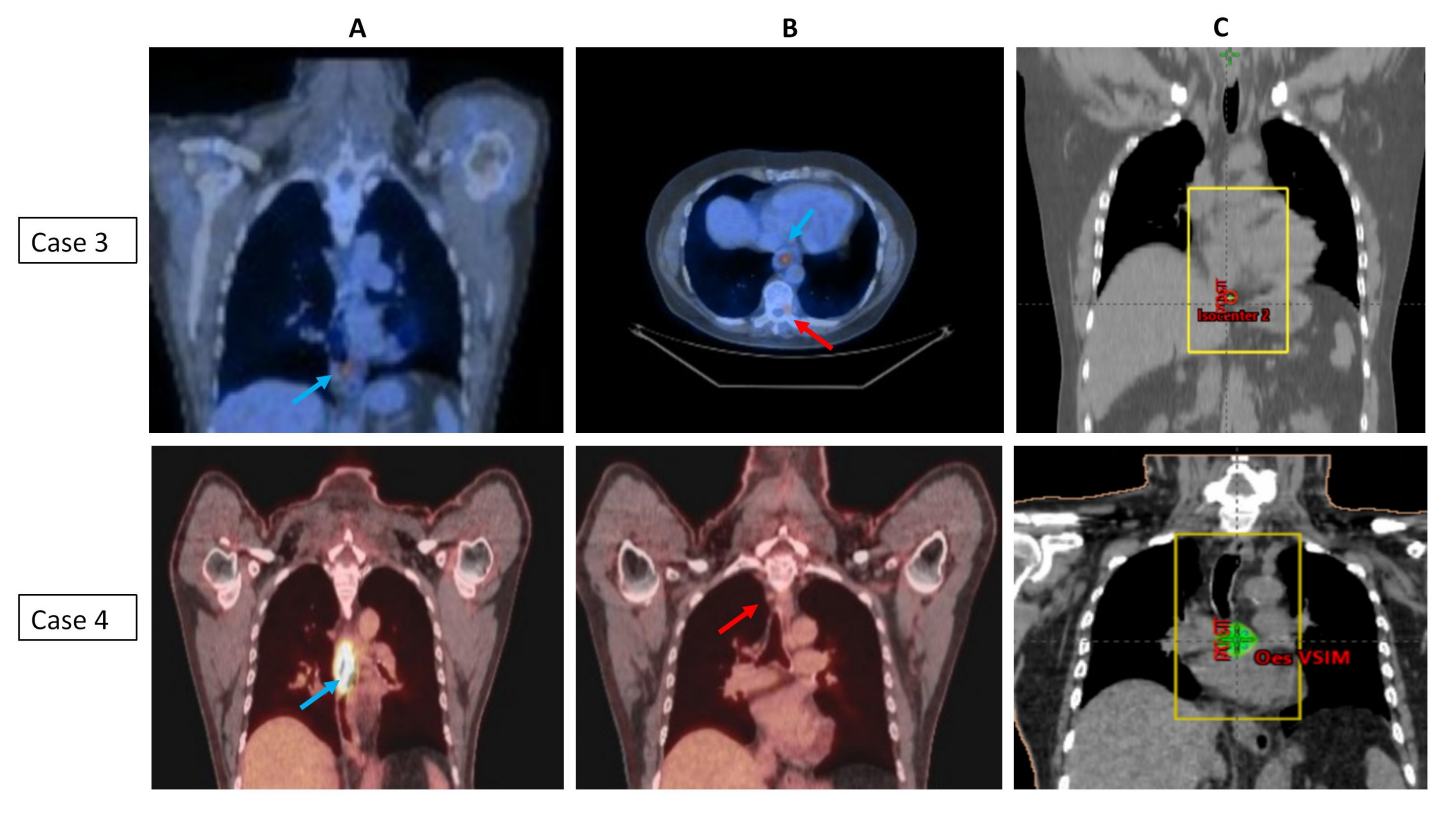
PET imaging showing the primary tumor and area of oligometastasis and corresponding radiotherapy portals (case 3 and case 4). (A) Coronal PET scans of cases 3 and 4; (B) axial and coronal PET scans of cases 3 and 4; (C) radiotherapy portals in cases 3 and 4. PET images with blue arrow indicating primary tumor and red arrow indicates site of oligometastasis (case 3: T9 vertebral metastasis; case 4: high paraesophageal node); radiotherapy portals were defined to cover the primary tumor and adjacent areas of metastatic disease/visible residual disease (metastasis-directed therapy)

All patients received a palliative dose of 30 Gy in 10 fractions and were set up using virtual simulation in supine position with arms by side and placement of two lateral and central anterior tattoos. The target definition included placement of anterior and posterior fields with incorporation of primary tumor and any adjacent areas of residual metastatic disease with 2–3 cm margin to account for any microscopic spread and organ motion/patient movement. Multi-leaf collimation was to shield any adjacent areas as clinically appropriate. Patients were prescribed 30 Gy in 10 fractions to the isocentre (parallel opposed pair of fields) and delivered 5 days a week over 2 weeks. Image verification was performed daily with kilovoltage (kV) imaging prior to each treatment. Patient staging and corresponding RT portals are illustrated in Figure 1 (case 1 and case 2) and Figure 2 (case 3 and case 4).

### Follow up

All patients were followed up with clinical assessments combined with regular CT scans performed at 3–6 monthly intervals. The CT scans showed no measurable disease in all cases consistent with complete response as per RECIST (Response Evaluation Criteria in Solid Tumors) criteria. In addition, cases 1 and 2 had follow up FDG-PET and OGD in 2017 after recommendation from the specialist MDT with the aim of offering salvage surgery if any residual disease had been identified. As no residual disease was identified, patients continued with routine follow-up. Case 4 had PET/CT done in 2018 to investigate non-specific esophageal thickening which showed no residual FDG uptake.

All cases are alive and well and have survived for more than 5 years (range = 7–13 years) from original diagnosis with no evidence of recurrence of esophageal malignancy. Case 2 was diagnosed with primary lung malignancy 8 years after original diagnosis and remains under appropriate oncological follow-up. Case 4 was diagnosed with prostate cancer 5 years after original diagnosis and received radical RT combined with androgen deprivation therapy (ADT) and is in biochemical remission.

The patient characteristics including presentation, investigations, treatment, follow-up and outcomes are summarised in Table 1.

**Table 1:** Table outlining the clinical summary of Cases (1–4), clinical presentation, diagnosis and staging, treatment, follow-up and present status

**Patient factors**	**Case 1**	**Case 2**	**Case 3**	**Case 4**
Demographics	49 years/Male	68 years/Male	53 years/Male	68 years/Male
Clinical presentation	Feb 2011 Dysphagia, weight loss	Feb 2013 Dysphagia	Feb 2010 Dysphagia	June 2015 Dysphagia
WHO performance status (PS) Comorbidities	PS0 No significant PMH	PS0 No significant PMH	PS0 No significant PMH	PS0 Type II DM, hypertension
Investigations	OGD—tumor 34–38 cm PET/CT—multiple loco-regional nodes, upper para-aortic lymphadenopathy, lung nodules	OGD—tumor 40–46 cm PET/CT—loco-regional gastro-hepatic node, para-aortic lymphadenopathy	OGD—tumor 35–40 cm (non-traversable) PET/CT—avid high left para-tracheal LN and T9 vertebral metastasis	OGD—tumor 28–35 cm EUS—high para-esophageal LN PET—avid high paraesophageal LN (outside surgical resection field)
Diagnosis staging	Intestinal type moderately differentiated AC Final staging T3N3M1	AC Final staging T3N1M1	AC Final staging T3N1M1	Squamous cell carcinoma (SqCC) T3N0M1 (non-regional LN)
Treatment	Apr 2011–Sep 2011—completed 6 cycles EOX Oct 2011—consolidation RT to primary tumor and adjacent nodal disease (gastro-hepatic): 30 Gy in 10 fractions	Jun 2013–Nov 2013—cycles cisplatin/capecitabine and trastuzumab Dec 2013—Consolidation RT adjacent nodal disease (gastro-hepatic and para-aortic): 30 Gy in 10 fractions Nov 2013–Apr 2017—maintenance trastuzumab	Feb 2010–Jul 2010—6 cycles EOX Aug 2010—Consolidation RT to primary tumor and adjacent area of metastatic disease (T9 vertebrae): 30 Gy in 10 fractions	Sep 2015–Dec 2015—5 cycles EOX chemotherapy Feb 2016—consolidation RT primary tumor and nodal disease: 30 Gy in 10 fractions
Follow-up	3–6 monthly interval CT scans Jun 2017—PET/CT and OGD	3–6 monthly interval CT scans Jul 2017—PET/CT and OGD	3–6 monthly interval CT scans Dec 2018—PET/CT	3–6 monthly interval CT scans
Present status (Mar 2023)	Alive and well No evidence of recurrence	Alive and well No evidence of recurrence	Alive and well No evidence of recurrence	Alive and well No evidence of recurrence

PMH: past medical history; DM: diabetes mellitus; EOX: epirubicin, oxaliplatin, and capecitabine; OGD: oral gastroduodenoscopy; EUS: endoscopic ultrasound; CT: computed tomography; PET: positron emission tomography; LN: lymph node; AC: adenocarcinoma

## Discussion

The European Society for RT and Oncology (ESTRO) and European Organisation for Research and Treatment of Cancer (EORTC) have developed a consensus classification and nomenclature of OMD as part of the Oligo-Care project. The classification introduced the concept of ‘induced OMD’ after systemic therapy in patients with previous poly-metastatic disease which was distinguished from ‘genuine OMD’ that was further subdivided into new onset ‘de novo’ OMD or ‘repeat’ OMD developing after previous treatment for OMD [6]. De-novo OMD was divided into metachronous (> 6 months after treatment of primary) and synchronous OMD (< 6 months after treatment of primary).

All the patients in the present case series presented with locally advanced EC and synchronous small-volume metastatic disease. Three patients (Case 2–4) had only 1–2 sites of distant metastasis (bone and/or non-regional LN) and one patient (case 1) had lung and non-regional LN involvement. In retrospect, all the above patients could have been classified as ‘genuine de novo’ OMD. They were treated with standard protocol of initial chemotherapy followed by RT to primary tumor which also included adjacent areas of visible/residual disease that could be safely included in the radiation field (metastasis-directed therapy) (Figures 1 and 2) [7].

In the recent past, several retrospective studies have reported on improved outcomes in EC patients with OMD. These studies have included patients presenting with synchronous or metachronous OMD and have reported 5-year survival rates of 25–50 percent. These studies have emphasised the use of appropriate local (plus systemic) therapy for treating the OMD lesions and adequate control of the primary tumor. These studies have employed different local treatment modalities including surgery with metastasectomy or RT with stereotactic ablative RT (SABR) or conventionally fractionated RT [8–14]. The importance of optimisation of local therapy was highlighted in a recent meta-analysis in which local therapy improved OS compared with systemic anti-cancer therapy alone (HR: 0.47, 95% CI: 0.30–0.74) including liver oligo-metastases (HR: 0.39; 95% CI: 0.22–0.59) [4].

All cases were treated with fractionated RT with palliative dose of 30 Gy in 10 fractions. As it was palliative dose patients were set up and treated with simple parallel opposed pair field arrangement as the dose was within cord tolerance. However, advances in RT technology now easily enable dose escalation with delivery of radical dose (50–60 Gy) to primary tumor with conventional (dose per fraction of 1.8–2 Gy) or hypofractionation (dose per fraction of > 2 Gy).

The advancement of imaging techniques enables better localization of the tumor volume. There are expert panel consensus guidelines available for contouring of target volumes in EC which recommend definition of gross tumor volume (GTV) using a combination of pre-treatment PET scan, CT scan, and endoscopy appearances (EUS preferable) [15]. The continuous advancements in RT technology over the past decades have allowed for EC to be treated with 3-dimensional (3D) treatment planning, including intensity-modulated RT (IMRT), volumetric-modulated arc therapy (VMAT) and helical tomotherapy. These modern techniques alongside advancements in image-guided RT allow the oncologist to deliver higher doses of radiation with more precision to the tumor and with less toxicity to the surrounding normal tissue, which has dramatically reduced morbidity. An additional consideration when defining target volumes is the effect of respiratory motion, which is most important for distal tumors and those involving the GOJ. Multiple techniques that control or account for respiratory motion are available and one of the most straightforward is the measurement of respiratory motion using 4D-CT at the time of simulation, in which case the magnitude of respiratory excursion can be incorporated into the choice of planning target volume (PTV) margin [16]. MRI-guided respiratory gated IMRT is another exciting technology that can be used to incorporate and adjust the organ and tumor motion within the contouring algorithm enabling improved sparing of adjacent organs at risk [17]. Proton therapy is particulate irradiation that deposits maximum dose in immediate vicinity of tumor with no ‘exit’ dose which can be potentially associated with an improvement in the therapeutic index by reducing dose to surrounding organs at risk. In phase 2 study comparing protons with IMRT the use of proton therapy was associated with significant reduction in toxicity burden and post-operative complications in patients with EC [18].

Liu et al. (2020) [19] reported on prospective, single-arm, phase 2 trial to assess the safety and efficacy of SABR for patients with OM SqCC of esophagus. The main inclusion criteria were 3 or fewer metastases and a controlled primary malignancy after radical treatment, with all metastatic lesions amenable to SABR. The 1- and 2-year overall survival rates were 76.2% and 58.0%, respectively [19]. Al-Batran et al. (2017) [20] reported on a prospective phase 2 randomized study (AIO-FLOT3) in patients with OM AC of the stomach and GOJ who received neoadjuvant chemotherapy (FLOT) prior to surgical resection. The median overall survival (OS) was 31.3 months (95% CI: 18.9–NA) for patients who proceeded to surgery [20].

Kroese et al. (2022) [12] reported on multicentre study of 205 patients with de-novo OMD (first-time diagnosis of ≤ 5 distant metastases on FDG-PET/CT). The majority of included patients had EC (73%) with AC histology (79%) and metachronous OMD (52%). The primary tumor was controlled in 68 percent. Improved OS was independently associated with combined use of local plus systemic therapy. The median OS after local plus systemic therapy was 35 months (95% CI: 22–NA) as compared with 13 months (95% CI: 9–21, *P* < 0.001) after systemic therapy alone [12].

The addition of systemic anti-cancer therapy (SACT) including chemotherapy and immunotherapy agents alongside local therapy has been shown to improve outcomes. However, the optimal timing and scheduling of SACT remains unknown. In the phase 2 study by Liu et al. (2020) [19] patients received chemotherapy after completion of SABR and in AIO-FLOT3 patients received neoadjuvant FLOT regime before surgery [20]. In one of the studies evaluating the use of PD1 inhibitors both sequential and concurrent approaches were used and found to be safe and effective.

In patients presenting with synchronous OMD neoadjuvant chemotherapy would seem to be the most appropriate initial option as it may help to select patients appropriate for subsequent local treatment. All the four patients in present case series with synchronous OMD were treated with upfront chemotherapy followed by RT to primary tumor and inclusion of adjacent areas of metastatic disease particularly all areas of visible residual disease. Therefore, the patients had both primary and metastasis-directed therapy.

These cases were identified retrospectively in view of the unexpected long-term survival and were rather peculiar as areas of residual metastatic disease after initial chemotherapy were adjacent to the site of primary tumor and could be included in the same radiation portal. However, the same may not be true for many patients with more distant sites of OMD where an alternative strategy for metastasis-directed therapy may be warranted possibly in the form of separate RT portals or SABR.

The importance of controlling the primary tumor in the context of OMD has been reported previously [11] and was highlighted in the recent phase III STAMPEDE and HORRAD trials in patients with de-novo hormone sensitive OM prostate cancer. A comprehensive meta-analysis of these studies revealed an OS benefit for prostate-directed RT in patients with fewer than 5 bone metastases (HR: 0.73; 95% CI: 0.58–0.92; *P* = 0.007) [21].

The dose of RT used in all patients was standard hypo-fractionated palliative regime of 30 Gy in 10 fractions which is less than the biological equivalent dose (BED) used in other studies. In a retrospective study of metachronous OMD in OG cancer 55 patients received RT to areas of metastasis. Patients receiving radical dose of RT (BED_10_ ≥ 60 Gy) had significant survival benefit compared to those receiving less than radical dose of RT (median OS of 16 months cf. 10 months, *P* = 0.033) [10]. Similarly, in another study of synchronous OMD there was an indication of better outcomes in patients with limited extra-regional LN involvement who could receive radical dosage of RT. All patients received more dose-intense schedules of chemotherapy compared to above studies which may have influenced the eventual complete response despite palliative dosage of RT. It also remains unclear whether the above responses were due to increased radio-sensitivity determined by particular molecular signature sub-types.

The combined approach using systemic and local therapy (including primary and metastasis-directed therapy) appears to be the favoured approach associated with the best outcomes and based on limited available data [12, 14, 19].

The unusual outcome of these patients renders further support to the existence of probable OM state in EC which may represent a completely different and prognostically favourable sub-group. It adds to the growing pool of evidence indicating the management strategy of OMD patients should be different from those with conventional metastatic disease. A combined strategy of local therapy (surgery or radiation) with appropriate control of primary and metastasis-directed therapy and systemic therapy may achieve best long-term outcomes. The performance of focused prospective research with large phase III RCTs to determine the optimal management of OMD in EC is absolutely essential. The OligoMetastatic Esophagogastric Cancer (OMEC) consortium was designed to develop a multidisciplinary consensus statement for the definition and treatment for OM esophagogastric cancer and drive the direction of future research [22, 23].

### Learning points

- OMD represents a stage of transition between localized and widely disseminated disease. The presence of OMD in OG cancer remains controversial.
- Retrospective studies and 2 prospective phase II studies have reported on improved outcomes in OG cancer with oligometastasis.
- We have presented 4 cases with EC and synchronous OMD who experienced long-term survival and cure after combined therapeutic strategy using systemic therapy followed by local RT targeting primary tumor and adjacent areas of residual metastatic disease (metastasis-directed therapy).

## Abbreviations

AC: adenocarcinoma
CI: confidence interval
CT: computed tomography
EC: esophageal cancer
EOX: epirubicin, oxaliplatin, and capecitabine
EUS: endoscopic ultrasound
FDG: fluoro-deoxyglucose
GOJ: gastroesophageal junction
IMRT: intensity-modulated radiotherapy
LN: lymph node
OG: oesophago-gastric
OGD: oral gastroduodenoscopy
OMD: oligometastatic disease
PET: positron emission tomography
PMH: past medical history
PS: performance status
RT: radiotherapy
SABR: stereotactic ablative radiotherapy
SqCC: squamous cell carcinoma

## Declarations

### Availability of data and materials

The raw data supporting the conclusions of this manuscript will be made available by the authors, without undue reservation, to any qualified researcher.

### Copyright

© The Author(s) 2024.
